# Enabling Efficient Folding and High-Resolution Crystallographic Analysis of Bracelet Cyclotides

**DOI:** 10.3390/molecules26185554

**Published:** 2021-09-13

**Authors:** Yen-Hua Huang, Qingdan Du, Zhihao Jiang, Gordon J. King, Brett M. Collins, Conan K. Wang, David J. Craik

**Affiliations:** 1 Institute for Molecular Bioscience, The University of Queensland, Brisbane, QLD 4072, Australia; y.huang@imb.uq.edu.au (Y.-H.H.); qingdan.du@imb.uq.edu.au (Q.D.); zhihao.jiang@uq.net.au (Z.J.); b.collins@imb.uq.edu.au (B.M.C.); c.wang@imb.uq.edu.au (C.K.W.); 2 Australian Research Council Centre of Excellence for Innovations in Peptide and Protein Science, The University of Queensland, Brisbane, QLD 4072, Australia; 3 The Centre for Microscopy and Microanalysis, The University of Queensland, Brisbane, QLD 4072, Australia; g.king@uq.edu.au

**Keywords:** peptides, crystal structures, cyclic peptides, cyclotides, quasi-racemic crystallography

## Abstract

Cyclotides have attracted great interest as drug design scaffolds because of their unique cyclic cystine knotted topology. They are classified into three subfamilies, among which the bracelet subfamily represents the majority and comprises the most bioactive cyclotides, but are the most poorly utilized in drug design applications. A long-standing challenge has been the very low in vitro folding yields of bracelets, hampering efforts to characterize their structures and activities. Herein, we report substantial increases in bracelet folding yields enabled by a single point mutation of residue Ile-11 to Leu or Gly. We applied this discovery to synthesize mirror image enantiomers and used quasi-racemic crystallography to elucidate the first crystal structures of bracelet cyclotides. This study provides a facile strategy to produce bracelet cyclotides, leading to a general method to easily access their atomic resolution structures and providing a basis for development of biotechnological applications.

## 1. Introduction

**Figure 1:**
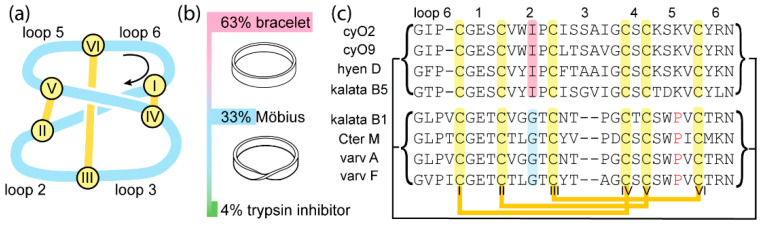
Cyclotide topology, relative abundance of subfamilies, and sequence alignment of selected cyclotides. (**a**) Illustration of the cyclic cystine knot (CCK) motif that defines cyclotide topology. The yellow circles represent cysteine residues, labelled with Roman numerals according to their sequential order. (**b**) Bar chart showing the relative abundance of each subfamily. (**c**) Sequence alignment; orange and black lines represent disulfide bonds and the circular backbone, respectively. Cysteine residues are highlighted in yellow. The Pro residue in loop 5 of Möbius cyclotides that adopts a *cis* conformation is in red. The third residue in loop 2 of each sequence is thought to be essential for in vitro folding and is highlighted in pink (bracelet) or blue (Möbius). CyO2 and cyO9 are cycloviolacin O2 and O9, respectively. CyO2 and kalata B1 are prototypical members of the bracelet and Möbius subfamilies. Trypsin inhibitor cyclotide sequences are omitted for clarity.

Cyclotides are a large family of topologically unique peptides characterized by a head-to-tail cyclic backbone and three interlocked disulfide bonds [1,2]. This combination of structural features forms a cyclic cystine knot (CCK, Figure 1a) motif and confers cyclotides exceptional stability [3]. Correct folding of their structures is thought to be essential for cyclotides to carry out their natural plant defense functions against insects [4] and pathogenic fungi [5], as well as many other bioactivities suited for applications in agriculture and medicine [2]. The CCK motif forms a rigid framework that is highly tolerant to amino acid substitutions, making cyclotides ideal structural scaffolds, whereby many peptide pharmacophores have been grafted into one of the six backbone loops to design stable drug leads [6,7,8].

Cyclotides are divided into three subfamilies: bracelet, Möbius, and trypsin inhibitor subfamilies (Figure 1b). Of these, bracelet cyclotides are the most common, representing around two-thirds of all cyclotides discovered so far. However, structure–activity relationship studies have been based almost exclusively on Möbius cyclotides [9,10] because of their more facile synthesis. The structural feature used to distinguish bracelet from Möbius cyclotides is a *cis*-Pro in loop 5, which is present only in Möbius cyclotides and renders the main chain with a 180° twist [11]. As more cyclotides are being discovered, additional differences are being observed between the subfamilies, for instance, in the sequences of loops 2, 3, and 5, as shown in Figure 1c.

Since cyclotides were first reported two decades ago, studies on bracelet cyclotides have been hampered by their low synthetic yields because of poor oxidative folding efficiency [12,13,14]. In the standard oxidative folding condition that is able to achieve complete folding of kalata B1 (kB1, the prototypical Möbius cyclotide) into the native state [15], cycloviolacin O2 (cyO2, the prototypical bracelet cyclotide) forms a heterogenous mixture of non-native species, with no native state achieved [14]. An optimized folding condition for cyO2 increased the yield to 40%, but this condition has not been tested on other bracelet cyclotides and is a complex detergent-containing mixture that is potentially unsuitable for use at industrial scale [14]. Thus, bracelet cyclotides are still regarded as being synthetically inaccessible, and, therefore, facile strategies to make them are still being sought.

Here, we provide a way of overcoming the synthetic hurdle of producing bracelet cyclotides and show how this method opens opportunities to better understand them and facilitate the development of cyclotides as drug scaffolds. As a first step, we investigated approaches to obtaining X-ray structures of bracelet cyclotides. The structures of bracelet cyclotides have been extensively studied by NMR [16,17], but X-ray crystallography can provide higher resolution and more conclusive data on structural features such as the disulfide bond connectivity and hydrogen bond network [18]. However, obtaining diffraction-quality crystals of cyclotides has been notoriously difficult until the recent emergence of racemic crystallography. This approach uses a mixture of an l-peptide and its mirror image to increase the chance of obtaining crystals compared to studying the l-form alone [19,20]. It was recently used to obtain an atomic resolution structure of the Möbius cyclotide kB1 [21]. By contrast, there has been no X-ray structure of any bracelet cyclotide because the difficulty of their chemical synthesis has precluded their production. 

In this study, we report a single mutation in loop 2 that enables bracelet cyclotides to fold efficiently in vitro. This discovery was inspired by an alanine mutagenesis study of kB1 that showed the third residue in loop 2 (Gly-12) is important for its in vitro folding as Gly can adopt the positive φ-angle efficiently compared to residues with a longer side chain [9]. We found that when the loop 2 Ile is mutated to Gly, bracelet cyclotides could be folded with high yield, albeit with loss of activity. When the Ile was mutated to Leu instead, bioactivity was retained. Using this I11L mutation, we synthesized all d-enantiomers of I11L mutants of bracelet cyO2 from *Viola odorata* and bracelet hyen D from *Hybanthus enneaspermus* [22,23]. These mutants were mixed with wild-type cyO2 and hyen D to determine atomic resolution structures by quasi-racemic crystallography.

## 2. Results and Discussion

### 2.1. Substitution of Ile-11 to Gly or Leu Increases Folding Yield of Bracelet Cyclotides

To explore the effect of substituting the Ile residue in loop 2 to Gly, we first introduced the mutation into cyO2, which is considered a prototypical bracelet cyclotide and is one of the most studied [14,24,25,26]. It is also the most common bracelet in the Violaceae plant family, one of the five plant families known to produce cyclotides [1,22,27,28,29]. Following standard cyclotide synthesis procedures, the [I11G]cyO2 mutant was assembled as a linear amino acid chain and its termini were ligated to produce the cyclic reduced form. The cysteine residues were then oxidized under the folding condition commonly used for kB1, which comprises a mixture of an alkaline buffer, isopropanol, and redox agents [15]. Remarkably, the point mutation of Ile to a smaller residue led to a significant improvement in folding yield from negligible amounts for cyO2 to over 50% for the mutant cyO2 (Table 1). 

This mutation was subsequently introduced into three other bracelet cyclotides, i.e., cycloviolacin O9 (cyO9), hyen D, and kalata B5 (kB5), to explore whether it might have broader utility for improving synthetic yields. These peptides were chosen because they all contain an Ile at position 11 and exhibit varying and increasing sequence differences compared to cyO2. Specifically, these differences are in loop 3 for cyO9, loops 2, 3, and 6 for hyen D, and loops 2, 3, 5, and 6 for kB5, as indicated in Figure 1 and Table 1. For all three bracelet cyclotides, the replacement of Ile-11 with Gly unequivocally increased folding yields. As detailed in Table 1, folding yields increased from low (8.2% for cyO9) or negligible amounts (hyen D and kB5) to over 50%. 

Since Ile-11 appears to be critical for folding of bracelet cyclotides, it was of interest to explore the capacity of this position to tolerate another branched residue, Leu. Surprisingly, the four [I11L] mutants exhibited yields from 13%–44%, which is lower than that of the [I11G] mutants but still substantially higher than that obtained from the native parent peptides (Table 1). A single folding condition was used to allow for a fair comparison of the effect of the Ile to Leu or Gly substitution across different cyclotides. Detailed oxidative folding traces showing conversion from reduced to oxidized states of all mutants are provided as Figure S1 in Supplementary Material. We confirmed the oxidized mutants could adopt the native structure by purifying them from the folding mixture (analytical HPLC traces displaying high purity, shown in Figure S2 in Supplementary Material) and then comparing their NMR chemical shifts to those of their native counterparts, as shown in Figure S3 in Supplementary Material. No significant Hα chemical shift perturbations were observed, demonstrating that all mutants adopted the correct fold. 

Overall, our analyses of the Gly and Leu mutants showed that significant increases in the folding yield compared to the wild-type sequences can be achieved. Based on our survey of four different bracelet cyclotides, we speculated that this single substitution might have broader utility. Indeed, the recently discovered bracelet cyclotide hedyotide B1 from *Hedyotis biflora*, which has a Leu residue in loop 2, has a folding yield of 48%. Although the folding buffer used in that study contains more isopropanol (70% *v*/*v*) than used here, it corroborates our findings [30]. Our results also suggest that the size and/or flexibility at residue 11 is a critical factor governing bracelet cyclotide folding. Gly residues are smaller and more conformationally flexible than Leu, and Leu is methyl γ-branched and, thus, more flexible than β-branched Ile, which might explain why the Gly mutants fold the most efficiently of the three sequences followed by the Leu mutants and the Ile-containing wild-type cyclotides.

### 2.2. Leu Mutants Are as Active as the Wild-Type Bracelet Cyclotides

Cyclotides are reported to exert their biological activities via membrane binding, particularly by selective binding to lipids containing phosphatidylethanolamine (PE) headgroups [31,32]. Therefore, to evaluate the functional impact of the single mutation on bracelet cyclotides, the membrane-binding ability of these variants towards PE-containing lipid bilayers was examined using surface plasmon resonance (SPR). The model membrane comprising 80% 1-palmitoyloleoyl-phosphatidylcholine (POPC) and 20% 1-palmitoyloleoyl-phosphatidylethanolamine (POPE) was used. Binding affinity was evaluated by the peptide-to-lipid maximum binding value (P/L max, mol/mol) and the peptide concentration required to achieve half-maximum binding at equilibrium (K_D_ value, μM). As shown in Table 2, all [I11G] mutants did not bind the model membrane at peptide concentrations up to 128 µM, except [I11G]hD, which showed an 18-fold weaker affinity to membranes compared to its parent peptide. The negative effect of the [I11G] bracelet cyclotide mutation on lipid binding was expected, as the mutation reduces surface hydrophobicity, a previously reported determinant of membrane binding [33,34]. The SPR dose-response curves and sensorgrams are shown in Figure S4 in Supplementary Material.

Contrary to the [I11G] results, the [I11L] mutants exhibited equipotent binding to the POPC/POPE (80:20) model membrane compared to their wild-type counterparts (Table 2). This result is consistent with the hypothesis that hydrophobicity is important for bracelet membrane binding. To further confirm the activity of these mutants, their cytotoxicity against HeLa cancer cells was evaluated along with wild-type bracelet cyclotides. As shown in Table 2, [I11L] mutants exhibited similar or slightly higher cytotoxicity compared to their parent cyclotides, whereas the [I11G] mutants had substantially reduced or no activity, which correlates well with their membrane binding. Of the four [I11G] mutants, the hyen D analogue had the highest activity, possibly because hyen D has one more hydrophobic residue in loop 3 compared to the other three cyclotides, which may contribute to its higher lipid-binding capacity (P/L max = 0.37) and help offset the hydrophobicity loss caused by the [I11G] mutation. The cytotoxicity dose-response curves of all mutants are shown in Figure S4. Overall, these results confirmed that the [I11L] mutants retain the biological properties of their native bracelet cyclotides. This is significant because it suggests that the [I11L] mutants could potentially replace wild-type cyclotides in agrochemical or therapeutic applications as the wild-type cyclotides are among the most potent nematocidal [35], anti-fungi [5], and anticancer cyclotides reported to date [22,36].

### 2.3. High-Resolution Structures of Bracelet Cyclotides Can Be Determined by Quasi-Racemic Crystallography

**Figure 2:**
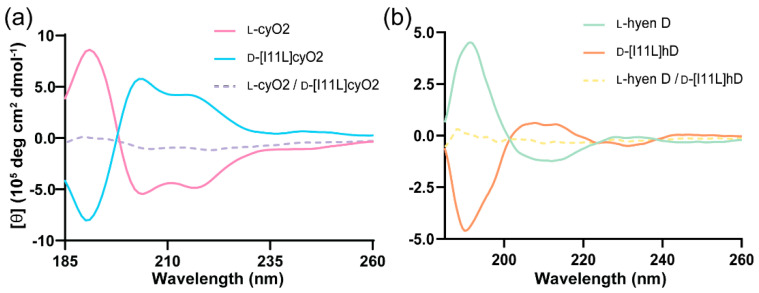
Circular dichroism (CD) spectra of the synthetic d-enantiomers and native (l-) bracelet cyclotides. (**a**) CD spectra of l-cyO2 (pink) and d-[I11L]cyO2 (blue) at 50 μM, and their mixture in equal ratio, in dashed line (purple). (**b**) CD spectra of l-hyen D (green) and d-[I11L]hD (orange) at 50 μM, and their mixture in equal ratio, in dashed line (yellow).

The ability to produce bracelet cyclotides, albeit single point mutants, at workable yields means we can now synthesize the d-isomers of bracelet mutants in a cost-effective way, which can facilitate structural determination of native bracelet cyclotides by quasi-racemic crystallography, a modification of racemic crystallography [20,21]. As reported previously, having the exact mirror image of a peptide is not essential to capture the benefits of a true racemic mixture in facilitating crystallization, and a near mirror image can often be used instead. Therefore, d-enantiomers of [I11L]cyO2 and [I11L]hD were chemically synthesized and used for quasi-racemic crystallization to determine structures of cyO2 and hyen D. The mirror-image symmetry of d-[I11L]cyO2 with l-cyO2 and d-[I11L]hD with l-hyen D was confirmed using circular dichroism (CD). As shown in Figure 2, the CD spectra of the d-enantiomers showed equal but opposite optical rotation to their corresponding native cyclotides despite being not exact mirror images in sequence. When the pair of peptides was mixed in equal ratio, the circular dichroism signals of both were neutralized by each other (dashed lines).

**Figure 3:**
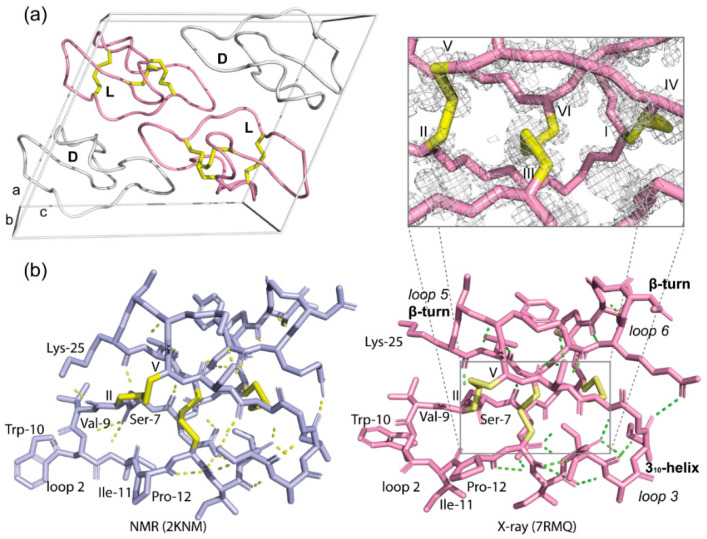
Quasi-racemic crystallography of cyO2. (**a**) Unit cell of quasi-racemate of wild-type cyO2 in space group P1 2_1_ 1. CyO2 is shown in pink and d-[I11L]cyO2 in white. (**b**) Comparison of cyO2 solution structure (light purple, PDB: 2KNM) and crystal structure (pink, PDB: 7RMQ). Yellow and green dashed lines represent the hydrogen bonds in the NMR and crystal structure, individually. Inset: 2Fo-Fc electron density map of the cystine knot shown at a σ level of 1.0.

The crystal structure of the quasi-racemate of wild-type cyO2 was solved in space group P1 2_1_ 1 at 1.17 Å resolution. The crystallization statistics are summarized in Table S1. Figure 3a shows the packing of l-cyO2 and d-[I11L]cyO2 in the unit cell, which contains two molecules of each peptide. The structure of cyO2 displays a small α-helix centered in loop 3 and a triple-strand β-sheet located in loops 4, 5, and 6 (Figure 3b). The inset of Figure 3b shows the disulfide connectivity of cyO2 is well defined by the electron density and confirms it forms the cystine knot topology, the structure of which is key to the ultrastability of cyclotides. 

Overall, the structure of cyO2 determined here using crystallography is similar to the solution structure previously determined by NMR spectroscopy (Figure 3b, left panel) [26], validating the quasi-racemic crystallography approach for structure elucidation of bracelet cyclotides. However, there are some notable differences. For instance, the II-V disulfide bond exhibited different conformations. Cystine residue conformations are considered more accurate in X-ray structures than those determined by NMR spectroscopy. The challenge in NMR spectroscopy is that methods to obtain experimental data on cystine residue dihedral angles can be subjective and time-consuming, leading to inaccuracies in the three-dimensional structure. This might explain why the hydrogen bond networks differ between the two cyO2 structures, with hydrogen bonds observed in the NMR structure but not in the crystal structure, e.g., between Lys-25 and Val-9. Another reason for these differences is because of sample artefacts, with the peptides being tightly packed in crystals used for X-ray structure determination but freely diffusing in solution in NMR spectroscopy. Despite these differences, it is clear the crystal structures provide complementary information to increase our understanding of the structures of bracelet cyclotides.

**Figure 4:**
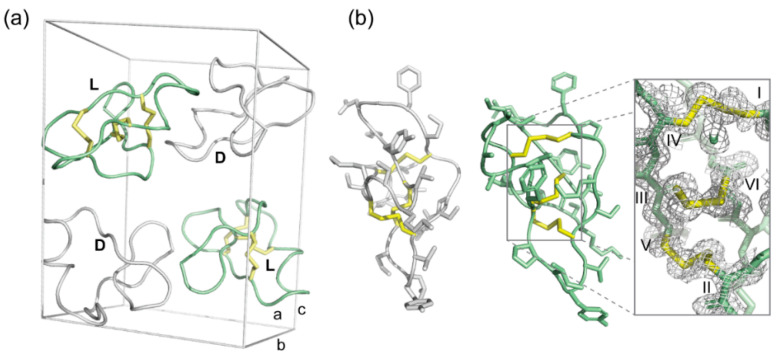
Quasi-racemic crystallography of hyen D (7RIH). (**a**) Unit cell of a quasi-racemate of wild-type hyen D in space group P1 2_1_ 1. Hyen D is shown in green and d-[I11L]hD in white. (**b**) Ribbon representation of hyen D (green) and d-[I11L]hD (white). Inset: 2Fo-Fc electron density map of the cystine knot shown at a σ level of 1.0.

We determined the crystal structure of another native bracelet cyclotide, hyen D, using the Ile-11 to Leu mutation and quasi-racemic crystallography to show the power of the approach. As shown in Figure 4a, the structure was solved in space group P1 2_1_ 1 and the unit cell comprises two pairs of hyen D and d-[I11L]hD. At 1.35 Å resolution, the structure is well defined, with the disulfide connectivity clearly confirmed to be that of the cystine knot (Figure 4b). Comparison of the crystal structures of hyen D (PDB ID: 7RIH) with cyO2 showed similarities in structure, including the presence of a short 3_10_ helix in loop 3 (Figure S5 from Supplementary Material). We also identified several residues that participate in conserved hydrogen bonds that stabilize the structure of bracelet cyclotides. For example, the second residue in loop 3, which is usually either a Ser or Thr, is involved in hydrogen bonds with residues within the same loop and with the sidechain of Glu in loop 1.

**Figure 5:**
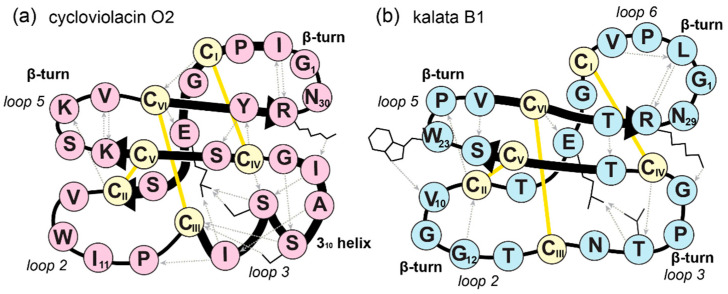
Comparison of the hydrogen bond network between cyO2 (this work, PDB ID: 7RMQ) and kB1 (PDB ID: 4TTM). Disulfide bonds are shown as yellow sticks. (**a**) Schematic representation of the structure and hydrogen bond network of cyO2. Secondary structures are highlighted as black arrows and ribbon. Hydrogen bonds are illustrated as gray dashed arrows. (**b**) Schematic representation of the structure and hydrogen bond network of kB1. Cysteine residues are labelled with Roman numerals in sequential order.

To gain more insight into the differences between bracelet and Möbius cyclotides and why they might have different folding yields, the crystal structures of cyO2 and kB1 were compared (Figure 5) [21]. Between the two peptides, the hydrogen bond network exhibited differences. Specifically, kB1 has more hydrogen bonds between different loops. We note, in particular, the hydrogen bond between Val-10 (loop 2) and Trp-23 (loop 5), for which an equivalent hydrogen bond to connect loop 2 to 5 is not present in cyO2. The presence of these additional hydrogen bonds might promote correct folding of kB1 compared to cyO2 by stabilizing intermediate states or reducing the folding free energy of the native state. The importance of the Trp-23 residue is supported by the previous site-directed mutagenesis study of kB1, which showed that folding yield decreased markedly after it was mutated to Ala [9].

### 2.4. Racemic and Quasi-Racemic Crystal Structures of Ile-11 Mutant Bracelet Cyclotides

**Figure 6:**
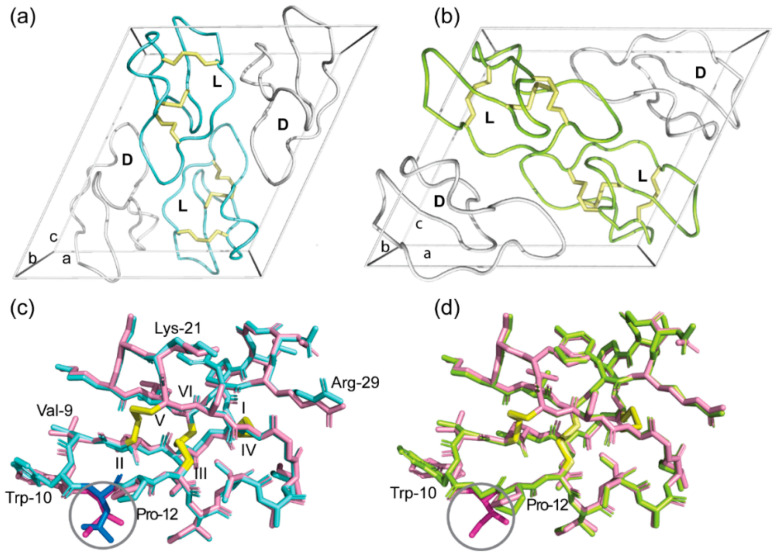
Racemic and quasi-racemic crystallography of [I11L]cyO2 (7RMR) and [I11G]cyO2 (7RMS). (**a**) Unit cell of the true racemate of [I11L]cyO2 in space group P1 2_1_ 1 with ribbon representations of the cyclic peptide molecules. The l-enantiomers are in blue and d-enantiomers in white. The disulfide bonds are shown as yellow sticks. (**b**) Unit cell of quasi-racemate of [I11G]cyO2 in space group P1 2_1_ 1. The [I11G]cyO2 is shown in lemon and d-[I11L]cyO2 in white. (**c**) Superimposition of cyO2 (pink) with [I11L]cyO2 (blue). The Ile and Leu residues in loop 2 are highlighted in the circle. (**d**) Superimposition of cyO2 (pink) with [I11G]cyO2 (lemon). The Ile and Gly residues in loop 2 are highlighted in the circle.

**Figure 7:**
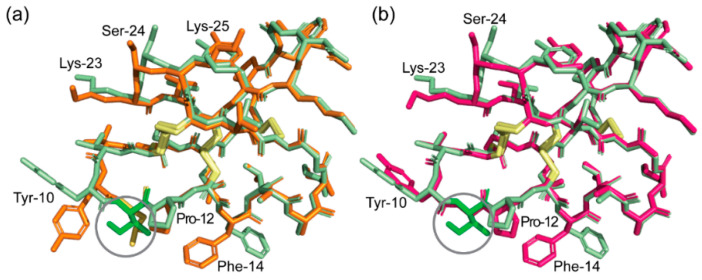
Racemic and quasi-racemic crystallography of [I11L]hD (7RII) and [I11G]hD (7RIJ). (**a**) Superimposition of hyen D (green) with [I11L]hD (orange). The Leu and Ile residues in loop 2 are highlighted in the circle. (**b**) Superimposition of hyen D (green) with [I11G]hD (hot pink). The Ile and Gly residues in loop 2 are highlighted in the circle.

Having determined the crystal structures of wild-type cyO2 and hyen D, we were interested in definitively showing their Ile-11 to Gly and Leu mutants have native-like structures to support our earlier NMR chemical shift analysis. Their structures were solved using racemic or quasi-racemic crystallography. Figure 6a,b shows the unit cells for the crystal structures of [I11L]cyO2 (PDB ID: 7RMR) and [I11G]cyO2 (PDB ID: 7RMS), respectively. Both structures align well with that of cyO2 (Figure 6c,d, respectively) and exhibit only minor differences in the backbone conformation of loop 2 and side chains of some residues, including Trp-10 and Pro-12. Figure 7a,b shows the crystal structures of [I11L]hD and [I11G]hD, respectively. The [I11L]hD (PDB ID: 7RII) was solved in space group Pī, while [I11G]hD (PDB ID: 7RIJ) was in P1 (Figure S6). Like the case of cyO2 mutants, [I11L]hD and [I11G]hD structures only exhibited minor differences in the mainchain and sidechain conformations, confirming both Leu and Gly substitutions are well tolerated by the bracelet fold.

## 3. Materials and Methods

### 3.1. Plant Materials and Cyclotide Isolation

*Hybanthus enneaspermus*, *Viola odorata,* and *Oldenlandia affinis* were grown in the glasshouse at the University of Queensland. Crude plant extracts were prepared in 50% MeCN (with 1% formic acid), filtered, and lyophilized. The extracts were fractionated using a Strata C18-E solid phase extraction (SPE) cartridge (with 20%, 50%, and 80% MeCN); the fraction eluted with 50% MeCN was collected and lyophilized. Hyen D (*H. enneaspermus*), cyO2 and cyO9 (*V. odorata*), and kB5 (*O. affinis*) were isolated from the 50% MeCN fractions using preparative and semi-preparative reversed-phase high-performance liquid chromatography (RP-HPLC) to >95% purity. The molecular integrity and purity of peptides were examined using LC/MS (LCMS-2020, Shimadzu, Kyoto, Japan). 

### 3.2. Selected Bracelet Cyclotides’ Synthesis and Folding

All cyclotide variants were synthesized using Fmoc-based solid-phase peptide synthesis, as described previously [37]. The linear precursors with sidechain protecting groups were assembled on 2-chlorotrityl chloride resin using an automated peptide synthesizer (Symphony^®^, Protein Technologies, Tucson, Arizona), and then were cleaved from the resin using 1% TFA in dichloromethane. The sidechain protected linear precursors were subsequently head-to-tail cyclized in solution using hexafluorophosphate azabenzotriazole tetramethyl uronium in the presence of N, N-diisopropylethylamine in dimethylformamide for 6 h. Finally, the sidechain deprotection was carried out in a mixture of triisopropylsilane:H2O:TFA (2:2:96, *v*/*v*/*v*) and stirred for 2.5 h. The crude cyclic peptides were purified via RP-HPLC and were folded under a set of folding conditions with a mixture of isopropanol and 0.1 M ammonium bicarbonate (pH 8.5) supplemented with 2 mM reduced glutathione and 0.8 mM oxidized glutathione. The correctly folded cyclotide variants were purified using RP-HPLC to >95% purity determined by analytical HPLC (Figure S2). The yield of peptides with native-like conformation was calculated by comparing the peak area of the folded peptide to the total area of reduced peptide obtained from analytical RP-HPLC. The final peptide quantities were 2.2, 2.7, 2.5, and 1.2 mgs for [I11L]cyO2, [I11L]cyO9, [I11L]hD, and [I11L]kB5 and 4.3, 5.8, 3.5, and 2.8 mgs for [I11G]cyO2, [I11G]cyO9, [I11G]hD, and [I11G]kB5, individually. 

### 3.3. Cytotoxicity Study 

The cytotoxicity of the peptides was evaluated using the resazurin assay, as described previously [22]. HeLa cells were seeded at 5000 cells/well in a 96-well, flat-bottomed plate one day prior to the experiments. Peptide solutions were prepared in water and incubated with HeLa cells at concentrations ranging from 16 to 0.125 μM or 8 to 0.063 μM in triplicate (final concentrations). Water and 1% Triton X-100 (TX) were applied as negative and positive control, respectively. After 24-h incubation, 10 μL of resazurin (0.05%, *w*/*v*) was added and incubated with cells for another 18 h. The absorbance was measured at 540 and 620 nm on a microplate reader (Infinite M1000 Pro microplate reader, Tecan, Switzerland). The percentage of cell death was quantified by calculating the absorbance ratio, R (R = absorbance at 620/540), and applying the following equation: % Cell death = (R_sample_ − R_H2O_)/(R_TX_ − R_H2O_) × 100. Data were analyzed with GraphPad Prism software (version 8.3.1) using Specific binding with Hill slope algorithm. 

### 3.4. Membrane-Binding Evaluation Using Surface Plasmon Resonance

The binding affinity of bracelet cyclotides and their variants toward lipid bilayers was studied using SPR, as described previously [38]. Two synthetic lipids, 1-palmitoyl-2-oleoyl-glycero-3-phosphocholine (POPC) and 1-palmitoyl-2-oleoyl-sn-glycero-3-phosphoethanolamine (POPE), were purchased from Avanti Polar Lipids (USA). Briefly, homogenous, small, unilamellar vesicles composed of POPC/POPE (80:20, mol/mol) were obtained by extruding the vesicle suspension through a 50-nm pore size polycarbonate membrane. L1 sensor chips and BIAcore T200 instrument were used (Cytiva, Marlborough, MA, USA). After the lipid was loaded and formed a bilayer membrane on the L1 chip, peptide solutions were injected over the lipid surface for 180 s and the dissociation was monitored for 600 s. Peptides were tested at concentrations ranging from 4–32 μM. Peptide-to-lipid molar ratio and K_D_ values were calculated by nonlinear regression using GraphPad Prism software (version 8.3.1). Peptide solutions and lipid vesicles were prepared in HEPES buffer (10 mM HEPES, 150 mM NaCl, pH 7.4, filtered) for SPR studies.

### 3.5. Circular Dichroism (CD)

CD spectra of cyO2, hyen D, and the d-enantiomers of [I11L]cyO2 and [I11L]hD were required in water at 50 μM with a 0.1-cm path length quartz cell at room temperature. CD spectra were recorded by accumulating five scans, from 185 to 260 nm using a CD spectro-polarimeter (Jasco J-810). The molar ellipticity was calculated using the following equation: [θ] = θ/(c × l × 10) (degrees. cm^2^. decimole^−1^), where θ is the value of ellipticity given by the instrument (millidegrees), c is the concentration of the cyclotide solution in molar units, and l is the optical path length of the cuvette in centimeters (0.1).

### 3.6. Peptide Crystallization 

Lyophilized pure peptides, l-cyO2, l- and d-[I11L]cyO2, l-[I11G]cyO2, l-hyen D, l- and d-[I11L]hD, and l-[I11G]hD, were dissolved in water. Six peptide mixtures were prepared as follows: a quasi-racemic cyO2 solution composed of l-cyO2 and d-[I11L]cyO2 (2 mg/mL); a racemic [I11L]cyO2 solution composed of l- and d-[I11L]cyO2 (2 mg/mL); a quasi-racemic [I11G]cyO2 solution composed of l-[I11G]cyO2 and d-[I11L]cyO2 (2 mg/mL); a quasi-racemic hyen D solution composed of equimolar amounts of l-hyen D and d-[I11L]hD (1 mg/mL); a racemic [I11L]hD solution composed of equimolar amounts of l- and d-[I11L]hD (1 mg/mL); and a quasi-racemic [I11G]hD solution composed of equimolar amounts of l-[I11G]hD and d-[I11L]hD (1 mg/mL). Crystallization screenings were performed in 96-well microplates containing commercially available screening kits, JCSG (Molecular Dimensions), Index and PEG/ion (Hampton Reasech), using hanging drop vapor diffusion method at 20 °C at the UQ ROCX diffraction facility. Equivolume mixtures of peptide and crystallization solutions were prepared using a Mosquito crystallization robot (SPT Labtech, Melbourn, Hertfordshire, UK) and were incubated and imaged in a RockImager 1000 (Formulatrix, Bedford, MA, USA). 

Diffraction-quality crystals of the aforementioned racemates were obtained under a range of conditions within 1 to 3 weeks. The quasi-rcemates and racemates of the same parent peptide crystallized efficiently and formed crystals under similar conditions. The following conditions were for the well-diffracting crystals from which we successfully obtained diffraction data. For the quasi-racemic cyO2 mixture, crystals were grown from 0.2 M magnesium formate dihydrate, 20% (*w*/*v*) PEG3350. For the racemic [I11L]cyO2 mixture, crystals were grown from 0.1 M sodium thiocyanate, 23% (*w*/*v*) PEG3350. For the quasi-racemic [I11G]cyO2 mixture, crystals were obtained from 0.1 M sodium thiocyanate, 27% (*w*/*v*) PEG3350. For racemic [I11L]hD mixture, crystals were obtained from 20% (*w*/*v*) PEG 3350. For the quasi-racemic hyen D mixture, crystals were grown from 0.07 M citrate (pH 2.3), 0.03 M Bis-tris propane (pH 9.7), and 16% (*w*/*v*) PEG3350. For quasi-racemic [I11G]hD mixture, crystals were grown from 0.2 M calcium acetate and 20% (*w*/*v*) PEG 3350. Crystals were harvested, snap-frozen, and stored in liquid nitrogen for data collection. 

### 3.7. Crystal Structure Determination

X-ray diffraction data were collected on the MX1 and MX2 microfocus beamlines at the Australian Synchrotron and recorded with a Dectris EIGER detector. All the data collected were indexed and integrated by AutoXDS and scaled using Aimless. Crystal structures of the bracelet cyclotides and their variants were solved by molecular replacement using Phaser with the NMR solution structure of cyO2 (PDB ID: 2KNM) as the initial model. Crystallographic structure refinements were performed using PHENIX suite. The refined models were manually rebuilt using Coot guided by Fo-Fc difference maps. Data collection and refinement statistics are summarized in Table S1. All structure images were generated using PyMOL. The final refined structures of cyO2 quasi-racemate, [I11L]cyO2 racemate, [I11G]cyO2 quasi-racemate, hyen D quasi-racemate, [I11L]hD racemate, and [I11G]hD quasi-racemate have been deposited in the Protein Data Bank with the following codes 7RMQ, 7RMR, 7RMS, 7RIH, 7RII, and 7RIJ, respectively.

## 4. Conclusions

We demonstrated efficient oxidative folding of bracelet variants with single mutations at residue Ile-11 using four different cyclotides. The in vitro folding yield of the [I11G] bracelet mutants was significantly higher than that of the parent peptides. The [I11L] mutants exhibited improved folding yields and retained the membrane binding ability compared to their wild-type counterparts. We propose these Leu mutants could be surrogates of the native peptides to be used in agricultural or therapeutic applications, in which cost-effective production is a major concern. Additionally, our study describes the crystal structures of bracelet cyclotides for the first time. Aided by the single point mutants, we have demonstrated the utility of racemic and quasi-racemic crystallography. Overall, this study offers an alternative and efficient approach for in vitro folding of bracelet cyclotides. As the bracelet subfamily provides the largest pool of candidates for pharmaceutical applications and leads for drug design of all three cyclotide subfamilies, our study provides a basis for further study of cyclotide structure and function and their future use as drug design scaffolds.

## Figures and Tables

**Table 1:** Sequence and folding yield (%) of selected bracelet cyclotides and their mutants.

Peptide Name	Amino Acid Sequence	Folding Yield (%)
cyO2	GIPCGESCVWIPCISSAIGCSCKSKVCYRN	negligible
[I11G]cyO2	GIPCGESCVWGPCISSAIGCSCKSKVCYRN	51.6
[I11L]cyO2	GIPCGESCVWLPCISSAIGCSCKSKVCYRN	30.8
cyO9	GIPCGESCVWIPCLTSAVGCSCKSKVCYRN	8.2
[I11G]cyO9	GIPCGESCVWGPCLTSAVGCSCKSKVCYRN	59.2
[I11L]cyO9	GIPCGESCVWLPCLTSAVGCSCKSKVCYRN	27.3
hyen D	GFPCGESCVYIPCFTAAIGCSCKSKVCYKN	negligible
[I11G]hD ^1^	GFPCGESCVYGPCFTAAIGCSCKSKVCYKN	57.3
[I11L]hD ^2^	GFPCGESCVYLPCFTAAIGCSCKSKVCYKN	44.7
kB5	GTPCGESCVYIPCISGVIGCSCTDKVCYLN	negligible
[I11G]kB5	GTPCGESCVYGPCISGVIGCSCTDKVCYLN	50.5
[I11L]kB5	GTPCGESCVYLPCISGVIGCSCTDKVCYLN	13.3

^1^ [I11G]hD = [I11G]hyen D. ^2^ The folding of [I11L]hyen D ([I11L]hD) was optimized to explore the best folding yield this mutant can achieve, whereas the other peptides were folded using the standard cyclotide folding condition (0.1 M ammonium bicarbonate, 50% isopropanol, 2 mM GSH, and 0.4 mM GSSG, pH 8.0).

**Table 2:** Cytotoxicity and membrane-binding properties of bracelet cyclotides and their *mutants*.

Peptide Names	POPC/POPE (80:20)		CC_50_^1^ on HeLa (μM) ^2^
	P/L Max (mol/mol)	K_D_ (μM)	
cyO2	0.28 ± 0.00	3.90 ± 0.11	1.05 ± 0.06
[I11G]cyO2	n.b. ^3^	n.a. ^4^	>64
[I11L]cyO2	0.31 ± 0.01	4.71 ± 0.17	0.95 ± 0.08
cyO9	0.30 ± 0.01	4.83 ± 0.17	2.35 ± 0.13
[I11G]cyO9	n.b.	n.a.	>64
[I11L]cyO9	0.31 ± 0.00	4.26 ± 0.06	1.06 ± 0.03
hyen D	0.37 ± 0.01	4.48 ± 0.17	0.85 ± 0.03
[I11G]hD	0.24 ± 0.01	81.0 ± 7.91	50.2 ± 4.61
[I11L]hD	0.36 ± 0.01	4.39 ± 0.50	0.70 ± 0.02
kB5	0.22 ± 0.00	5.02 ± 0.17	3.11 ± 0.20
[I11G]kB5	n.b.	n.a.	>64
[I11L]kB5	0.22 ± 0.00	4.95 ± 0.12	4.31 ± 0.48

^1^ CC_50_: half maximum cytotoxic concentration; ^2^ data are mean ± SEM; ^3^ n.b., no binding was observed at tested concentrations; ^4^ n.a., not available.

## Data Availability

The crystal structures of bracelet cyclotides and their mutants presented in this study are openly available in Protein Data Bank (22 September 2021, https://www.rcsb.org/).

## Supplementary Materials

The following are available online, Figure S1. Oxidative folding traces of native bracelet cyclotides and their I11L and I11G mutants. Figure S2. The purity and mass spectra of synthetic peptides analyzed using LC/ESI-MS. Figure S3. Secondary Hα chemical shift comparison of Ile-11 mutants to their native bracelet cyclotides. Figure S4. Membrane binding and cytotoxicity of bracelet cyclotides and their mutants. Figure S5. Structural comparison of cyO2 and hyen D. Figure S6. Unit cells of [I11L]hD and [I11G]hD. Table S1. Summary of crystallographic data and refinement statistics.
