# Supplementary material for: Enabling Efficient Folding and High-Resolution Crystallographic Analysis of Bracelet Cyclotides
Source: Molecules. 2021 Sep 13;26(18):5554. doi: 10.3390/molecules26185554 (PMC8467136; doi:10.3390/molecules26185554)
Supplement: Supplementary file 1 [file molecules-26-05554-s001.zip › molecules-1356273-supplementary.pdf]

molecules

Supplemetary Materials

Enabling Efficient Folding and High-Resolution Crystallographic Analysis of Bracelet Cyclotides

Yen-Hua Huang [1,2,†], Qingdan Du [1,2,†], Zhihao Jiang [1,2], Gordon J. King [3], Brett M. Collins [1], Conan K. Wang [1,2] and David J. Craik [1,2,*]

[1] Institute for Molecular Bioscience, The University of Queensland, Brisbane, QLD 4072, Australia; y.huang@imb.uq.edu.au (Y.-H.H.); qingdan.du@imb.uq.edu.au (Q.D.); zhihao.jiang@uq.net.au (Z.J.); b.collins@imb.uq.edu.au (B.M.C.); c.wang@imb.uq.edu.au (C.K.W.)
[2] Australian Research Council Centre of Excellence for Innovations in Peptide and Protein Science, The University of Queensland, Brisbane, QLD 4072, Australia
[3] The Centre for Microscopy and Microanalysis, The University of Queensland, Brisbane, QLD 4072, Australia; g.king@uq.edu.au
* Correspondence: d.craik@imb.uq.edu.au; Tel.: +61-7-3346-2019
† These authors contributed equally to this work.

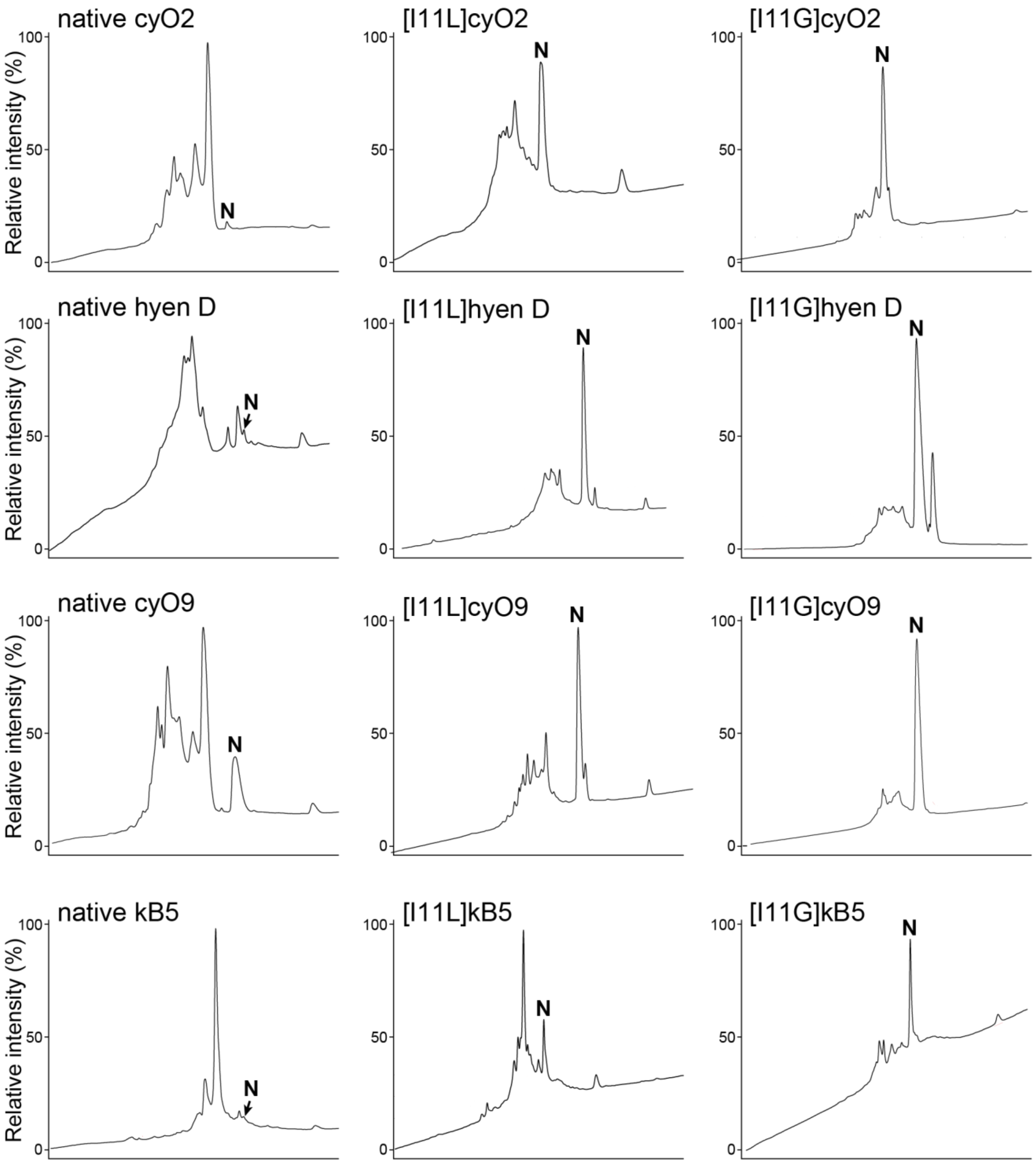

Figure S1. Oxidative folding traces of native bracelet cyclotides and their I11L and I11G mutants. 'N' represents peptide peaks with the native-like conformation.

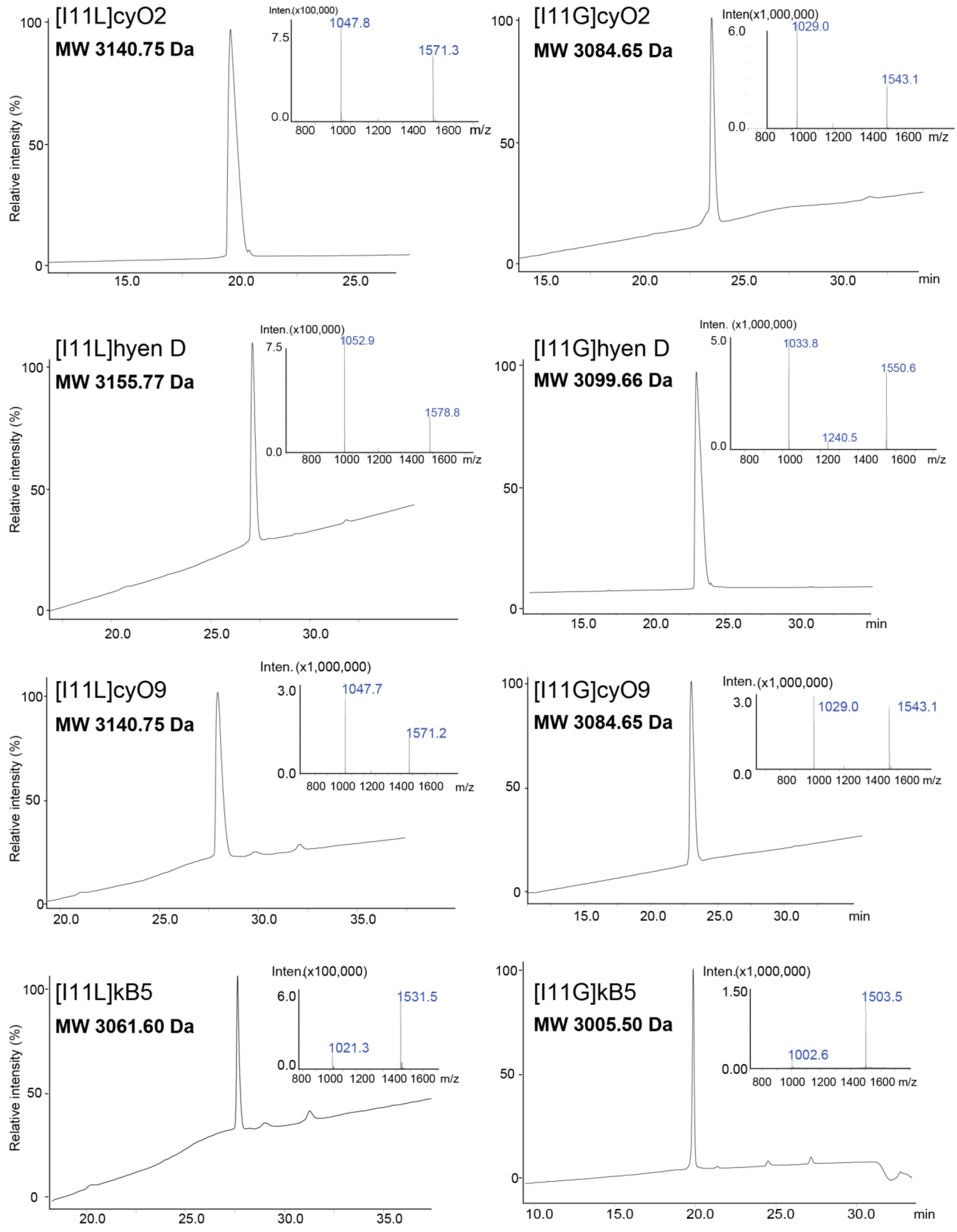

Figure S2. The purity and mass spectra of synthetic peptides analyzed using LC/ESI-MS.

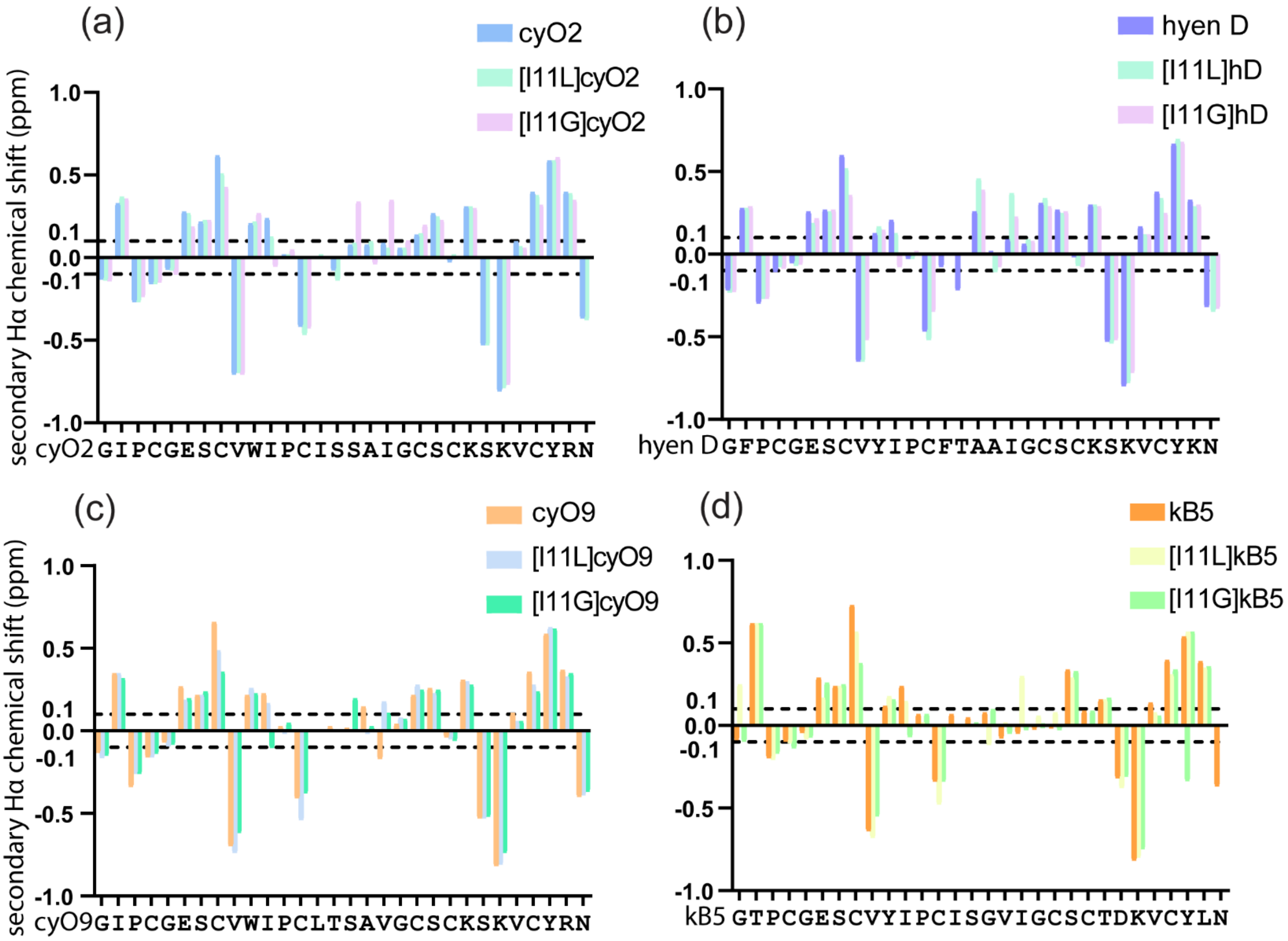

Figure S3. Secondary Hα chemical shift comparison of Ile-11 mutants to their native bracelet cyclotides hyen D (a), cyO2 (b), cyO9 (c), or kB5 (d) individually. Dashed lines indicate 0.1 and -0.1 ppm.

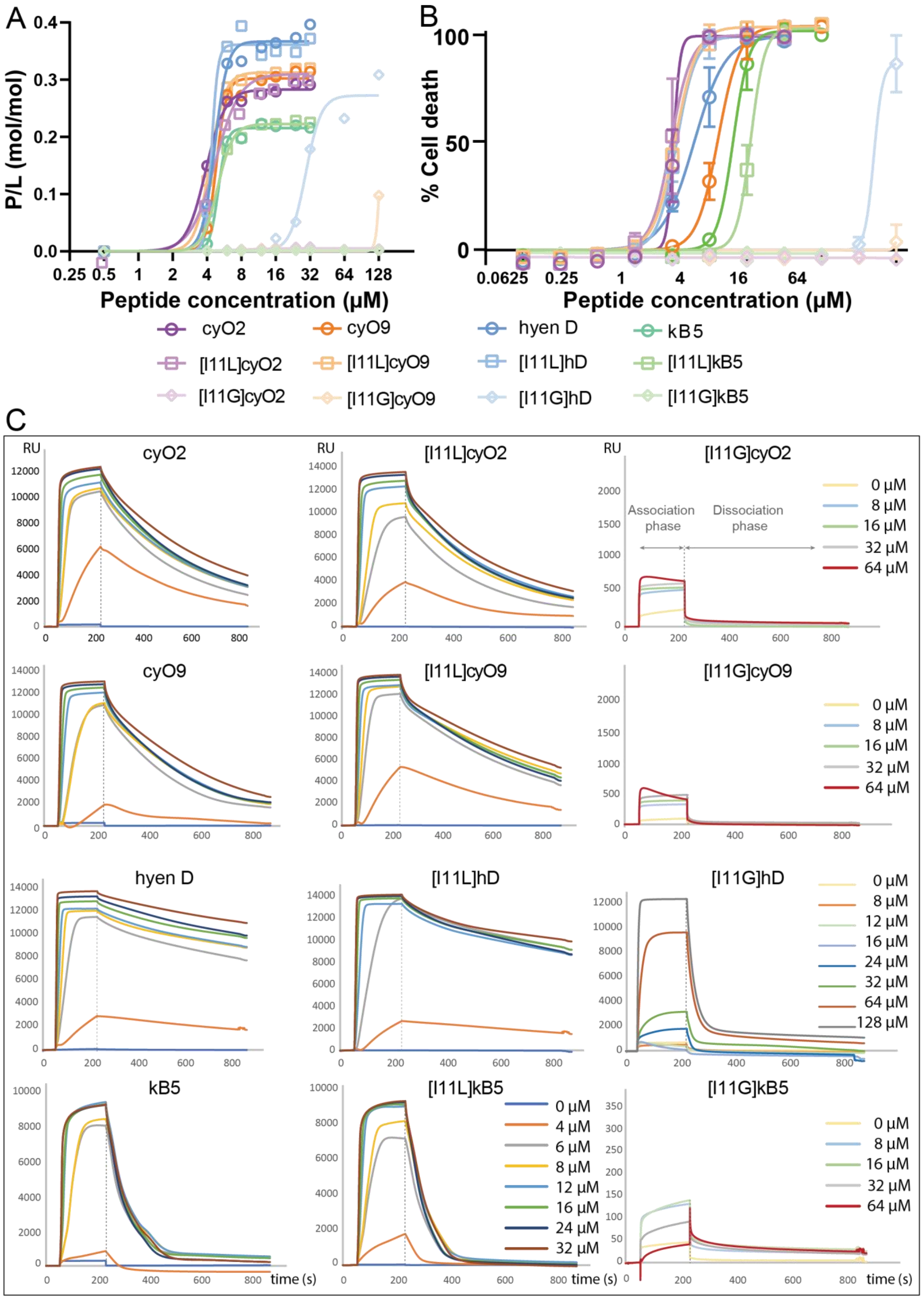
A
P/L (mol/mol)
Peptide concentration (µM)
B
% Cell death
Peptide concentration (µM)
cyO2
cyO9
hyen D
kB5
[I11L]cyO2
[I11L]cyO9
[I11L]hD
[I11L]kB5
[I11G]cyO2
[I11G]cyO9
[I11G]hD
[I11G]kB5
C
RU
cyO2
[I11L]cyO2
[I11G]cyO2
Association phase
Dissociation phase
cyO9
[I11L]cyO9
[I11G]cyO9
hyen D
[I11L]hD
[I11G]hD
kB5
[I11L]kB5
[I11G]kB5
0 µM
4 µM
6 µM
8 µM
12 µM
16 µM
24 µM
32 µM
64 µM
128 µM
time (s)

Figure S4. Membrane binding and cytotoxicity of bracelet cyclotides and their mutants. a) Concentration-response curves of cyO2, cyO9, hyen D, kB5 and their variants against the model membrane (POPC/POPE, 80/20) evaluated using SPR. b) The cytotoxicity of cyO2, cyO9, hyen D, kB5 and their variants evaluated against HeLa cells. c) Representative sensorgrams of cyclotides and their mutants obtained from serial diluted analyte concentrations. The gray dashed line marks the end of analyte injection/the beginning of dissociation phase.

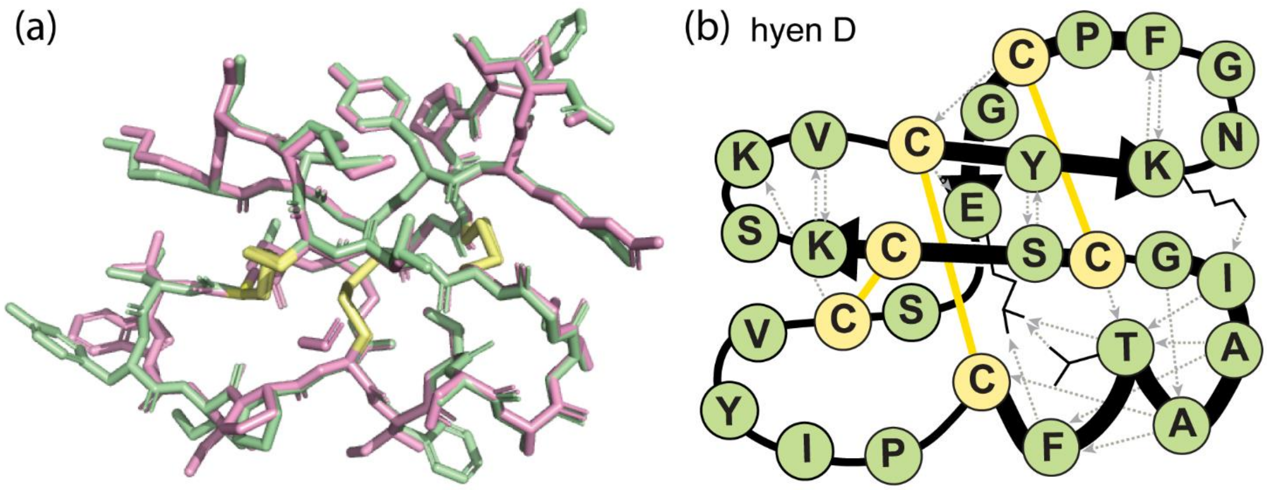

Figure S5. Structural comparison of cyO2 and hyen D. a) Superimposition of cyO2 (pink) with hyen D (green). b) The hydrogen bond network of hyen D.

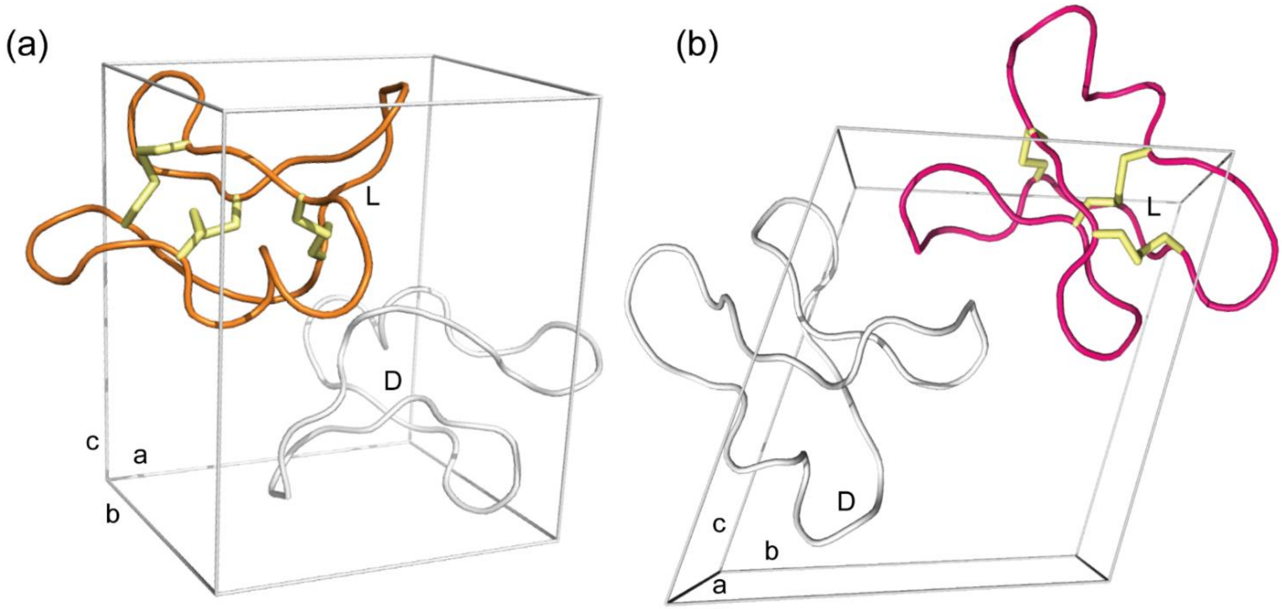

Figure S6. Unit cells of [I11L]hD and [I11G]hD. a) Unit cell of the true racemate of [I11L]hD in space group P$\bar{1}$ with ribbon representations of the cyclic peptide molecules. The L-enantiomer is shown in orange and the D-enantiomers in white. The disulfide bonds are shown as yellow sticks. b) Unit cell of quasi-racemate of [I11G]hD in space group P1. [I11G]hD is shown in hot pink and D-[I11L]hD in white.

Table S1. Summary of crystallographic data and refinement statistics*.

	cyO2 quasi-racemate	[I11L]cyO2 racemate	[I11G]cyO2 quasi-racemate	Hyen D quasi-racemate	[I11L]hD racemate	[I11G]hD quasi-racemate
**Data Collection**						
**Space group**	P 1 2_1 1	P 1 2_1 1	P 1 2_1 1	P 1 2_1 1	P $\bar{1}$	P 1
**Unit cell dimensions (a,b,c) (Å)**	28.00<br>24.74<br>29.91	27.10<br>24.76<br>30.24	27.29<br>25.11<br>29.93	26.038<br>24.731<br>34.729	18.755<br>21.153<br>26.296	22.655<br>23.85<br>24.246
**Unit cell angles (α,β,γ) (º)**	90.00<br>113.9<br>90.00	90.00<br>115.2<br>90.00	90.00<br>115.7<br>90.00	90.00<br>102.467<br>90.00	87.25<br>82.94<br>89.13	65.582<br>66.173<br>76.034
**Wavelength (Å)**	0.95370	0.95372	0.96410	0.95366	0.95372	0.97626
**Resolution range (Å)**	27.35-1.17 (1.21-1.17)	24.51-1.04 (1.08-1.04)	24.11-1.10 (1.14-1.10)	18.51-1.35 (1.40-1.35)	21.13-1.22 (1.264-1.22)	20.73-1.30 (1.35-1.30)
**Total reflections**	25265 (2431)	33274 (3303)	29798 (2939)	30296 (2790)	38367 (3922)	17843 (1790)
**Unique reflections**	12686 (1232)	17453 (1715)	14958 (1474)	9116 (883)	11130 (1091)	9743 (954)
**Multiplicity**	2.0 (2.0)	1.9 (1.9)	2.0 (2.0)	3.3 (3.2)	3.4 (3.6)	1.8 (1.9)
**Completeness (%)**	98.82 (96.77)	98.98 (97.89)	99.6 (99.9)	93.70 (91.04)	93.26 (90.46)	93.85 (89.39)
**Mean I/σ(I)**	20.46 (7.73)	6.47 (2.08)	19.0 (9.80)	13.11 (4.60)	5.09 (2.14)	17.04 (10.71)
**R-merge**	0.013 (0.057)	0.040 (0.225)	0.016 (0.038)	0.050 (0.239)	0.129 (0.440)	0.029 (0.053)
**R-meas**	0.018 (0.080)	0.056 (0.318)	0.023 (0.054)	0.060 (0.287)	0.154 (0.521)	0.042 (0.076)
**R-pim**	0.013 (0.057)	0.040 (0.225)	0.016 (0.038)	0.033 (0.157)	0.0827 (0.276)	0.029 (0.053)
**CC(1/2)**	1.000 (0.995)	0.998 (0.912)	0.999 (0.996)	0.998 (0.975)	0.989 (0.889)	0.998 (0.994)
**Refinement**						
**R-work**	0.2117 (0.2298)	0.1783 (0.2173)	0.153 (0.131)	0.181 (0.247)	0.205 (0.315)	0.146 (0.158)
**R-free[a]**	0.2317 (0.2582)	0.1980 (0.2565)	0.176 (0.158)	0.215 (0.297)	0.223 (0.361)	0.163 (0.210)
**Number of non-hydrogen atoms**	470	476	481	543	260	509
**Number macromolecule atoms**	430	430	426	437	223	430
**Number water atoms**	34	40	46	80	32	67
**RMS[b] bonds (Å)**	0.009	0.014	0.018	0.007	0.008	0.008
**RMS angles (º)**	1.40	1.59	1.78	1.15	1.18	1.42
**Ramachandran favored (%)**	85.71	92.86	92.86	92.86	89.29	96.43
**Ramachandran allowed (%)**	14.29	7.14	7.14	7.14	10.71	3.57
**Ramachandran outliers (%)**	0.00	0.00	0.00	0.00	0.00	0.00
**Clashscore**	2.40	0.00	4.87	0.00	0.00	0.00
**Molprobity score**	1.15	0.95	1.47	0.95	1.07	0.74

**Average B-factor**	8.30	8.37	8.20	12.93	9.17	8.21

*Statistics for the highest-resolution shell are shown in parentheses

[a]R-free, R-work with 10% of the experimental observations sequestered before refinement

[b]RMS, Root mean square
